# Inhibition of renin-angiotensin system attenuates type I alveolar epithelial cell necroptosis in rats after hyperbaric hyperoxic exposure

**DOI:** 10.3389/fmed.2025.1521729

**Published:** 2025-02-21

**Authors:** CuiHong Han, PeiXi Zhang, Ying Liu, JiaJun Xu, XuHua Yu, YuKun Wen, ShiFeng Wang, WenWu Liu

**Affiliations:** ^1^Department of Pathology, Jining No 1 People’s Hospital, Jining, Shandong, China; ^2^Department of Cardiothoracic Surgery, Jining No 1 People’s Hospital, Jining, Shandong, China; ^3^Department of Pathology, Yantaishan Hospital, Yantai, Shandong, China; ^4^Department of Diving and Hyperbaric Medicine, Naval Medical Center, Shanghai, China

**Keywords:** hyperbaric hyperoxic lung injury, necroptosis, renin-angiotensin system, type I alveolar epithelial cells, inflammation

## Abstract

**Objective:**

There is evidence showing both necroptosis and activation of renin-angiotensin system (RAS) are involved in the pathogenesis of hyperbaric hyperoxic lung injury (HLI). This study aimed to investigate whether RAS activation can induce lung cell necroptosis and the cell specificity of necroptosis in the lung in case of hyperbaric HLI.

**Methods:**

Male SD rats were randomly assigned into control group (*n* = 12), HLI group (*n* = 18), captopril group (*n* = 18), and valsartan group (*n* = 18). Rats were pre-treated with intraperitoneal captopril (50 mg/kg) or intragastrical valsartan (30 mg/kg) for 3 days before hyperbaric exposure. Then, animals were exposed to 99.9% oxygen at 250 kPa for 6 h to induce HLI. After hyperbaric exposure, lung function was non-invasively detected, and then animals were sacrificed for the detection of wet to dry ratio of the lung, blood gas and lung inflammatory factors, and lung tissues were collected for double immunofluorescence staining. Statistical analysis was performed with one way analysis of variance.

**Results:**

Either valsartan or captopril pre-treatment could inhibit lung edema, improve blood gas (0 h) and lung function (48 h), and reduce pro-inflammatory factors in the lung. In addition, valsartan or captopril pre-treatment could inhibit AGT1 expression and lung cell necroptosis, and type I alveolar epithelial cells (AECs) were the major cell type experiencing necroptosis after hyperbaric hyperoxic exposure.

**Conclusion:**

Our study indicates inhibition of RAS can suppress the hyperbaric HLI, which may be, at least partially, related to the inhibition of type I AECs necroptosis. Our findings provide new mechanism for the protective effects of RAS inhibition on hyperbaric HLI.

## 1 Introduction

It has been confirmed that long term exposure to hyperoxic environment is toxic to human body (1). When the oxygen partial pressure is higher than 50 kPa (but lower than about 200 kPa), persistent exposure to this hyperoxic environment may cause damage to the lung, which is also known as pulmonary oxygen toxicity. In routine clinical practice, premature neonates are exposed to hyperoxic environment as a treatment, but it is a normobaric hyperoxic environment (2). There is high-quality evidence supporting that liberal oxygen exposure is associated with a dose-dependent increased risk in short-term and long-term mortality (3). However, in diving, divers are often exposed to a hyperbaric hyperoxic environment (4). There is evidence showing that the mechanism of hyperoxic lung injury (HLI) after normobaric hyperoxic exposure is different from that after hyperbaric hyperoxic exposure (5).

To date, numerous studies have been conducted to investigate the mechanisms underlying the pathogenesis of HLI, and some mechanisms have been proposed (1). The renin-angiotensin system (RAS) is a peptidergic system with endocrine characteristics, and involved in the regulation of blood pressure and fluid balance (6). Some studies have indicated that RAS is closely related to the pathogenesis of some lung diseases such as acute lung injury (ALI), chronic obstructive pulmonary disease (COPD), and asthma (7). In our previous study, results also showed hyperbaric hyperoxic exposure could activate the RAS in rats, which was related to the pathogenesis of prolonged exposure to hyperbaric hyperoxic lung injury (8, 9).

There is evidence showing that cell death plays an important role in the pathogenesis of HLI (10). Our previous study indicated that oxidative stress induced necroptosis, a type of programmed cell death, was involved in the pathogenesis of HLI, and inhibition of necroptosis was protective against HLI (11). In recent years, studies have shown that angiotensin II, an important part of RAS, may activate necroptosis in some disease models (12). However, whether angiotensin II can induce necroptosis in case of HLI has not been reported so far. In this study, the relationship between angiotensin and necroptosis and the cell specificity of necroptosis were investigated in a rat model of hyperbaric hyperoxic exposure.

## 2 Materials and methods

### 2.1 Animals and grouping

A total of 66 Sprague Dawley rats weighing 200–220 g were purchased from Shanghai SLAC Experimental Animal Co., Ltd. This study was approved by the Naval Medical Center of Naval Medical University. Animals were randomly divided into control group (*n* = 12), HLI group (*n* = 18), captopril group (*n* = 18), and valsartan group (*n* = 18). This study was approved by the Ethics Committee of Naval Medical Center.

### 2.2 Establishment of animal model

Animals were housed for a week in a humidity and temperature control environment. They were placed in a small chamber which was then flushed with 99.9% oxygen for 5 min until the oxygen concentration in the chamber was higher than 96%. Then, the chamber was pressurized with 99.9% oxygen to 250 kPa within 3 min. Soda lime was placed at the bottom to remove carbon dioxide, the chamber was ventilated continuously with 99.9% oxygen, and the oxygen concentration in the chamber was continuously monitored and maintained at >96% during the hyperbaric exposure. Animals received hyperbaric exposure at 250 kPa for 6 h and then the chamber was depressurized to atmospheric pressure (100 kPa) within 5 min. In the control group, animals were placed in the chamber which was flushed with room air, but it was not pressurized.

### 2.3 Treatments

Animals in the captopril group were intraperitoneally treated with captopril (50 mg/kg) for 3 days before hyperbaric exposure (13). In the valsartan group, animals were orally treated with valsartan (30 mg/kg) for 3 days before hyperbaric exposure (14). In the HLI group, animals were intraperitoneally treated with normal saline.

### 2.4 Detection of lung function

Lung function was detected with a whole-body plethysmograph (WBP-8R; TOW-INT Tech, Shanghai, China). Briefly, the rat was allowed to stay in the detection chamber for at least 5 min until the parameters remained stable. The parameters of lung function were detected automatically and stored in Excel for further analysis. Detection was done at least three times and average was obtained. The parameters included inspiration time (TI), expiration time (TE), peak inspiratory flow (PIF), peak expiratory flow (PEF), respiratory frequency (f), tidal volume (Vt), and minute ventilation (MV).

### 2.5 Blood gas analysis

Rats were intraperitoneally anesthetized with 3% sodium pentobarbital (40 mg/kg) at specific time points. Arterial blood was collected from the abdominal aorta and added to the test cartridge. Blood gas analysis was done with a blood analyzer (i-STAT blood analyzer, Abbott, CA, USA). The parameters included the pH value, carbon dioxide partial pressure (PCO_2_), oxygen partial pressure (PO_2_), extracellular fluid base excess (BEecf), bicarbonate radical (HCO_3_^–^), total carbon dioxide (TCO_2_), oxygen saturation (SO_2_), hemoglobin (Hb) and hematocrit (Hct).

### 2.6 Detection of wet to dry ratio

Immediately after hyperbaric exposure, rats were intraperitoneally anesthetized with 3% sodium pentobarbital (40 mg/kg), and the lung tissues were collected and weighed. Then, the lung tissues were dried in an oven and weighed until the weight remained stable (3 days later). The wet to dry ratio was calculated as follow: (wet weight – dry weight) / dry weight × 100%.

### 2.7 Enzyme linked immunosorbent assay

Immediately after hyperbaric exposure, animals were intraperitoneally anesthetized with 3% sodium pentobarbital (40 mg/kg), and the lung was harvested and processed for enzyme linked immunosorbent assay (ELISA) with corresponding kits (interleukin-1β [IL-1β; EK301B] and tumor necrosis factor α [TNF-α; EK382HS]; Multi Sciences, China).

### 2.8 Western blotting

Lung tissues were collected and stored at −80°C for further use. In brief, lung tissues were homogenized in lysis buffer and then centrifuged. The supernatant was collected and the protein concentration was determined by using BCA method. Then, total proteins (20 mg) were loaded for separation on 15% SDS-polyacrylamide gel electrophoresis and transferred onto polyvinylidene fluoride (PVDF) membranes (Bio-Rad, USA). The membranes were blocked with 5% non-fat milk, followed by incubation with primary antibodies at 4°C overnight: AGT1 (Affinity, DF4910, 1:1000), RIP1 (Bioss, bs-5805R, 1:1000), RIP3 (Bioss, bs-3551R, 1:1000) and MLKL (Abcam, ab243142, 1:1000). The secondary antibody was HRP conjugated goat anti-rat GAPDH (1:10000). The images of protein bands were captured and the band density was analyzed with Image J software (NIH, Bethesda, MD, USA).

### 2.9 Pathological scoring

After hyperbaric exposure, animals were anesthetized and thoracotomy was performed. Animals were perfused with normal saline via the left ventriculus and then with 4% paraformaldehyde. Lung tissues were collected, fixed in 4% paraformaldehyde, embedded in paraffin and sectioned. Lung sections were processed for HE staining, and then observed under a light microscope. Pathological scoring was based on the alveolar congestion, alveolar hemorrhage, and infiltration or aggregation of neutrophils in the alveolar space or vascular wall, alveolar wall thickening, and/or hyaline membrane formation. These pathological features were independently scored using a four-point scale: 1 indicates normal and 4 represents the most severe injury.

### 2.10 Immunohistochemistry

In brief, lung sections (4 μm) were processed for HE staining and immunofluorescence staining. Surfactant C (SPC) (Proteintech 10774-1-ap; 1:1000) was used to stain type II alveolar epithelial cells (AEC), aquaporin 5 (AQP5) (ab315856; 1:2000) was employed to stain type I AEC and MLKL was used to mark necroptotic cells. In brief, lung sections were deparaffinized and rehydrated in gradient alcohol. After antigen retrieval, sections were treated with 2.5% normal serum and then with anti-MLKL/SPC/AQP5 antibody at 4°C, overnight. Sections were rinsed with PBS, and then treated with fluorescent secondary antibody. In negative controls, sections were incubated with PBS instead of primary antibody. Finally, sections were observed under a fluorescence microscope.

### 2.11 Statistical analysis

Statistical analysis was performed with GraphPad Prism 8 (GraphPad Software, Boston, MA, USA). Data are expressed as mean ± standard deviation and compared with one way analysis of variance (ANOVA) among groups, followed by Tukey’s *post hoc* test. A value of *P* < 0.05 was considered statistically significant.

## 3 Results

### 3.1 Effects of captopril and valsartan on the wet to dry ratio and lung structure after hyperbaric exposure

**FIGURE 1:**
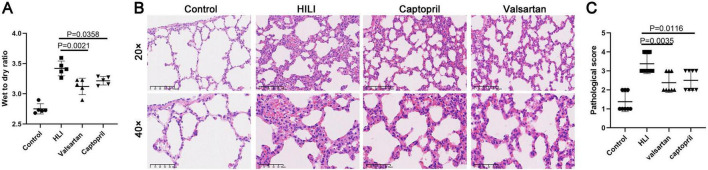
Wet to dry ratio and lung histological examination. **(A)** Wet to dry ratio immediately after hyperbaric hyperoxic exposure; **(B)** HE staining of lung tissues in different groups. **(C)** Histological scores of lung microstructure in different groups. ANOVA was used for comparisons among groups followed by Tukey’s *post hoc*.

Animals were sacrificed immediately after hyperbaric exposure and lung tissues were collected for the detection of wet to dry ratio. Our results showed both captopril and valsartan pre-treatment could reduce the wet to dry ratio of the lung tissues (*P* = 0.0021 and 0.0358; Figure 1A), but there was no significant difference between two treatment groups. As shown in Figure 1B, the lung structure was significantly damaged after hyperoxic exposure, which was characterized by the thickening of alveolar septum, alveolar hemorrhage, infiltration of inflammatory cells and rupture of alveolar septum. In addition, both captopril and valsartan pre-treatment attenuated the lung injury secondary to hyperoxic exposure which was manifested as the improvement of pathological scores (Figure 1C).

### 3.2 Effects of captopril and valsartan on the blood gas after hyperbaric exposure

**FIGURE 2:**
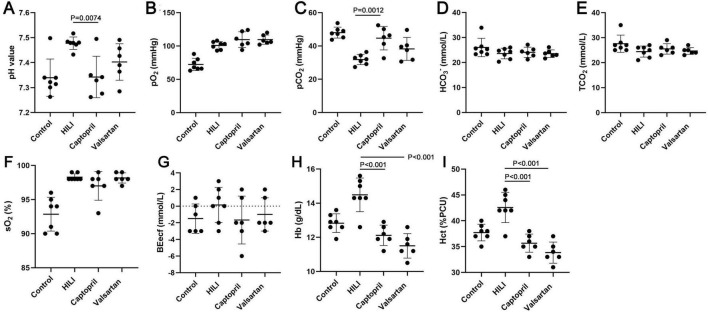
Blood gas in different groups immediately after hyperbaric exposure. **(A)** pH value; **(B)** oxygen partial pressure (PO_2_); **(C)** carbon dioxide partial pressure (PCO_2_); **(D)** bicarbonate radical (HCO_3_^–^); **(E)** total carbon dioxide (TCO_2_); **(F)** oxygen saturation (SO_2_), **(G)** extracellular fluid base excess (BEecf); **(H)** hemoglobin (Hb); **(I)** hematocrit (Hct). ANOVA was used for comparisons among groups followed by Tukey’s *post hoc*.

The blood gas was detected immediately after hyperbaric exposure. Our results showed the pH value, SO_2_, PO_2_, BEecf, Hb and Hct increased, but PCO_2_ reduced after hyperbaric exposure (Figure 2). In addition, captopril pre-treatment significantly reduced the pH value, Hb and Hct and markedly increased PCO_2_, and BEecf slightly reduced after captopril treatment; valsartan pre-treatment reduced pH value, BEecf, Hb and Hct, but increased PCO_2_, and significant difference was noted in Hb and Hct.

### 3.3 Effects of captopril and valsartan on the lung function after hyperbaric exposure

**FIGURE 3:**
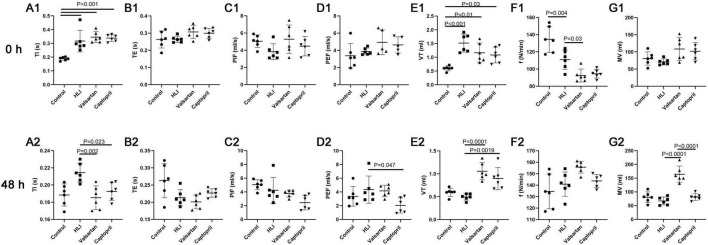
Lung function in different groups at 0 and 48 h after hyperbaric exposure. **(A1,A2)** Inspiration time (TI); **(B1,B2)** expiration time (TE); **(C1,C2)** peak inspiratory flow (PIF); **(D1,D2)** peak expiratory flow (PEF); **(E1,E2)** tidal volume (VT); **(F1,F2)** respiratory frequency (f); **(G1,G2)** minute ventilation (MV). ANOVA was used for comparisons among groups followed by Tukey’s *post hoc*.

In this study, lung function was non-invasively detected after hyperbaric exposure. TI and Vt increased significantly and the respiratory frequency reduced markedly after hyperbaric exposure. However, either captopril or valsartan pre-treatment failed to improve the lung function immediately after hyperbaric exposure except for the respiratory frequency. Therefore, the lung function was further detected at 48 h after hyperbaric exposure. Results indicated captopril pre-treatment significantly reduced TI and markedly increased Vt; valsartan pre-treatment significantly reduced TI and PEF and markedly increased Vt and MV at 48 h after hyperbaric exposure (Figure 3).

### 3.4 Effects of captopril and valsartan on the inflammation after hyperbaric exposure

**FIGURE 4:**
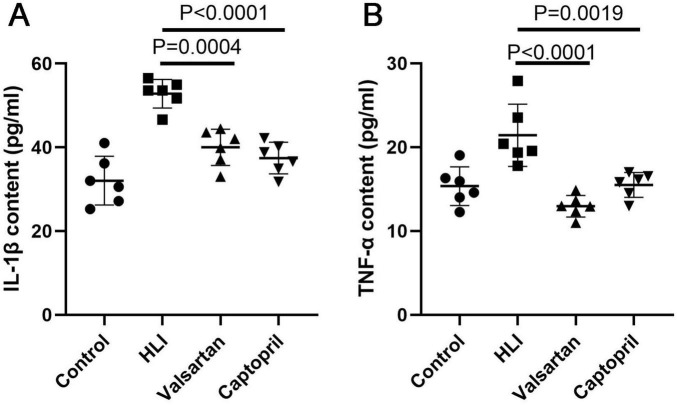
Contents of pro-inflammatory factors in the lung of different groups immediately after hyperbaric exposure. **(A)** IL-1β content of the lung; **(B)** TNF-α content of the lung. ANOVA was used for comparisons among groups followed by Tukey’s *post hoc*.

Inflammation is involved in the pathogenesis of HLI. In this study, we detected the pro-inflammatory factors IL-1β and TNF-α in the lung after hyperbaric exposure. Our results indicated the contents of IL-1β (*P* < 0.001) and TNF-α (*P* < 0.001) increased significantly in the lung after hyperoxic exposure (Figure 4). However, either captopril or valsartan pre-treatment could significantly inhibit the production of pro-inflammatory factors in the lung (*P* = 0.0004 and *P* < 0.0001, respectively), but there were no marked differences in the contents of IL-1β and TNF-α in the lung between captopril group and valsartan group (*P* > 0.05; Figure 4).

### 3.5 Cell-specific necroptosis in the lung after hyperbaric exposure

**FIGURE 5:**
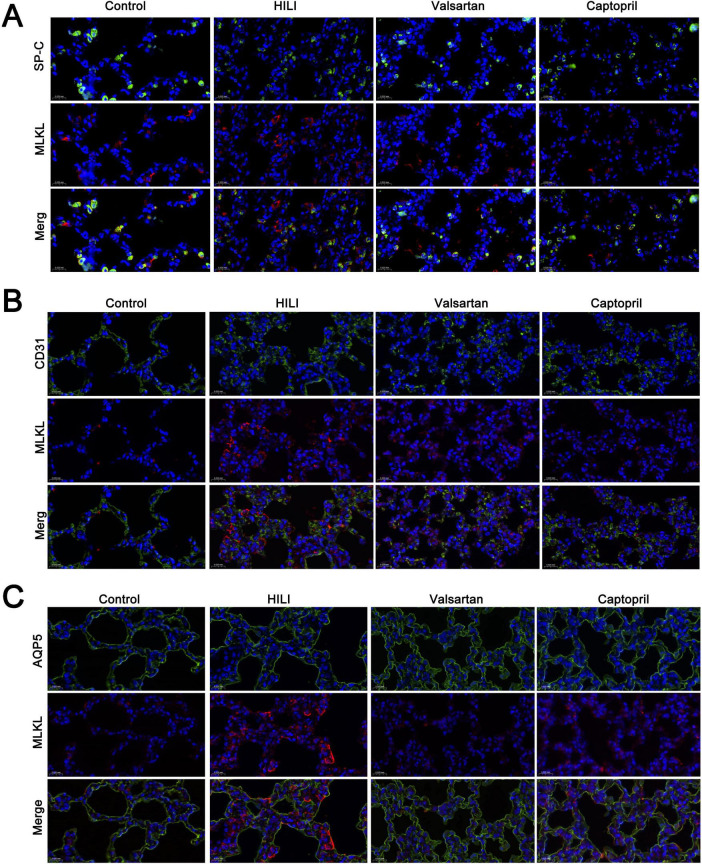
Cell-specificity of necroptosis in the lung. **(A)** Double immunofluorescence staining of type II alveolar epithelial cells (SP-C) and necroptosis (MLKL); **(B)** double immunofluorescence staining of blood vessels (CD31) and necroptosis (MLKL); **(C)** double immunofluorescence staining of type I alveolar epithelial cells (AQP5). Magnification: 40×.

Although our previous study showed necroptosis was involved in the pathogenesis of HLI, but the cell-specificity of necroptosis is still unclear. In the present study, double immunofluorescence staining was employed to investigate the cell-specificity of necroptosis in the lung. MLKL was used to indicate necroptosis. Our results showed most type II AECs and endothelial cells were negative to MLKL (Figures 5A, B), but type I AECs were positive to MLKL (Figure 5C). The number of MLKL positive cells increased significantly after hyperbaric exposure as compared to the control group, but either captopril or valsartan pre-treatment could markedly reduce the number of MLKL positive cells in the lung as compared to the HLI group (Figure 5C).

### 3.6 Protein expression of AGT1, MLKL, RIP1 and RIP3

**FIGURE 6:**
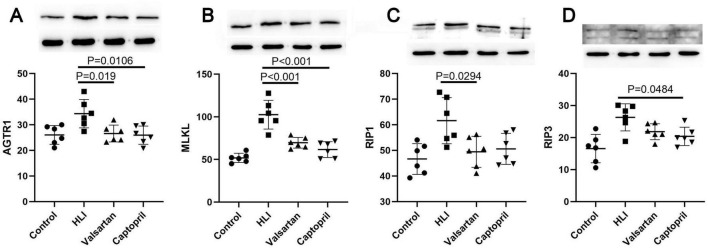
Expression of AGTR1 and necroptosis related molecules in different groups (Western blotting). **(A)** AGTR1 protein expression; **(B)** MLKL protein expression; **(C)** RIP1 protein expression; **(D)** RIP3 protein expression. ANOVA was used for comparisons among groups followed by Tukey’s *post hoc*.

Our results showed AGT1 expression increased significantly in the lung after hyperbaric exposure, but either captopril or valsartan pre-treatment could markedly reduce the expression of AGT1 in the lung (*P* = 0.019 and *P* = 0.0106, respectively; Figure 5A). In the lung, the protein expression of MLKL, RIP3 and RIP1 increased markedly after hyperoxic exposure. However, either captopril or valsartan pre-treatment decreased the protein expression of MLKL, RIP3 and RIP1 in the lung as compared to the HLI group (Figures 6B–D).

## 4 Discussion

In recent years, RAS has been found to possess pro-inflammatory, hypertrophic, pro-fibrotic, and pro-oxidative activities, and the dysregulations in the RAS is involved in the pathogenesis of many diseases, including lung diseases (7). Our previous study has indicated that the angiotensin II and angiotensin-converting enzyme (ACE) increased in the lung after hyperbaric HLI (8), which suggests the activation of RAS in the lung after hyperbaric HLI. It has been shown that cell death is involved in the pathogenesis of HLI and RAS activation may induce necroptosis, a type of programmed cell death (10, 12). Our group for the first time found that necroptosis is involved in the pathogenesis of HLI because inhibition of necroptosis is able to attenuate lung injury secondary to hyperbaric hyperoxic exposure (11). However, whether the activation of RAS can induce necroptosis in the lung in case of hyperbaric HLI has not been reported. In this study, we investigated the relationship between RAS activation and necroptosis in the lung after hyperbaric HLI. Valsartan and captopril were used to independently inhibit RAS activation. Valsartan is an angiotensin II receptor blocker (ARB) and can block angiotensin II from binding to the AT1 receptor in many tissues. Captopril is an ACE inhibitor (ACEI) and can suppress the conversion of angiotensin I into angiotensin II in tissues.

In the present study, animals were pre-treated with valsartan or captopril for 3 days before the hyperbaric hyperoxic exposure, and the protective effects were first evaluated by the wet to dry ratio, lung function and blood gas. Wet to dry ratio is a common indicator of lung edema. Wet to dry ratio analysis in the present study showed both valsartan and captopril could suppress lung edema secondary to hyperbaric hyperoxic exposure. The lung is responsible for gas exchange and thus lung injury is easy to cause the change in blood gas. Therefore, blood gas analysis is often employed to evaluate the severity of lung injury. In the present study, arterial blood was collected for blood gas analysis, and results showed both valsartan and captopril could improve the changes in blood gas after hyperbaric exposure: significantly reduced pH value, Hb and Hct, and slightly reduced BEecf, but markedly increased PCO_2_ after hyperbaric hyperoxic exposure. Lung injury is often translated to the change in lung function, and lung function detection can identify the mild lung injury if the clinical symptoms are not evident. In diving medicine, the reduction in tidal volume is closely related to the HLI (15). There is evidence showing that hyperoxic exposure could increase airway resistance and decrease pulmonary compliance (16). To further understand the protective effects of valsartan and captopril on the lung function, non-invasive lung function detection was performed immediately after hyperbaric exposure. However, the lung function largely remained unchanged immediately after hyperbaric exposure (except for respiratory frequency). Therefore, lung function was detected at 48 h after hyperbaric exposure. Increase in TI (increased resistance of inspiration) and decrease in respiratory frequency (deep breath) were the most common changes in the lung function after hyperbaric exposure and both valsartan and captopril could significantly reduce TI and increase respiratory frequency after hyperbaric exposure. In addition, both valsartan and captopril increased Vt, leading to the elevation of MV after hyperbaric exposure. These indicate valsartan and captopril pre-treatment may improve the lung function. All these findings indicate both valsartan and captopril (inhibition of RAS) are able to inhibit the lung injury secondary to prolonged exposure to hyperbaric hyperoxic environment.

It has been reported that lung cell death (including apoptosis, necrosis, autophagy and pyroptosis) plays an important role in the pathogenesis of HLI (17–19). In our previous study, results indicated necroptosis was involved in the pathogenesis of hyperbaric HLI (11). However, whether the necroptosis is cell-specific is still unclear. In this study, double immunofluorescence staining was employed to investigate the cell specificity of necroptosis in lung tissues after hyperbaric hyperoxic exposure. Type I ACEs are the most common type of cells in the lung and responsible for the gas exchange in the lung. In addition, they serve as the first-line defense against environment and therefore are susceptible to the damage due to the environmental change. In our study, immunofluorescence staining not only indicated the necroptosis increased after hyperbaric hyperoxic exposure, but also type I ACEs were found to be the major cells experiencing necroptosis secondary to hyperoxic exposure, and few type II ACEs and endothelial cells were necroptotic in the lung. After hyperoxic exposure, there was an evident loss of healthy capillary endothelial cells in the lung (20), which was different from our findings. This might be ascribed to the difference in the animal model.

In 2006, Bhandari et al reported that hyperoxia could cause angiopoietin 2-mediated acute lung injury and necrotic cell death (21). Whether inhibition of RAS may inhibit the necroptosis in the lung is poorly understood. Immunofluorescence staining and Western blotting were employed to detect the expression of major proteins in the necroptosis process. Both detections confirmed the expression of necroptosis related proteins reduced after hyperbaric hyperoxic exposure in case of valsartan or captopril pre-treatment. Further examination indicated either valsartan or captopril pre-treatment significantly reduced the contents of pro-inflammatory factors in the lung after hyperbaric hyperoxic exposure. A study on the normobaric hyperoxic exposure indicated inflammatory activation is the major driver of the observed transcriptional changes in hyperoxia (20). These findings suggest either valsartan or captopril can inhibit cell necroptosis and subsequent inflammation in the lung, leading to the improvement of lung edema, lung function and blood gas. However, the exact mechanism underlying Ang II induced necroptosis is still unclear. It has been reported that the binding of Ang II to its receptor can induce the increase in intracellular calcium (22, 23), and the increased intracellular calcium may further induce RIP1/RIP3 complex-dependent necroptosis (24). Whether intracellular calcium mediates the Ang II induced necroptosis is warranted to be further elucidated in more studies.

Taken together, the novelty of this study was that our group for the first time investigated the relationship between RAS activation and necroptosis in the lung after hyperbaric hyperoxic exposure and further explored the cell-specificity of necroptosis in case of HLI. Our results show hyperbaric hyeroxic exposure mainly induces the necroptosis of type I AECs, and inhibition of RAS with valsartan or captopril before exposure can effectively prevent the lung against hyperbaric HLI. Both valsartan and captopril have pleiotropic activities and have been widely used in the clinical treatment of diseases with favorable safety. Thus, both valsartan and captopril have the promise to become a preventive strategy for hyperbaric HLI. However, there were still limitations in this study. In our previous study, the blood gas was analyzed immediately and at 48 h after hyperbaric hyperoxic exposure, and results showed the blood gas nearly returned to the levels in the control group at 48 h after hyperbaric exposure. Thus, in this study, blood gas at 48 h after exposure was not detected. As previously reported, valsartan and captopril can exert some bioeffects beyond their primordial role in the cardiovascular system, and the signaling pathway by which valsartan and captopril inhibit lung cell necroptosis is still unclear and was not further explored in this present study. In our study, the dose dependent response was not investigated for valsartan and captopril although protective effects of valsartan and captopril were indicated, and thus the optimal dose at which preventive effect is achieved with few adverse effects is still unclear. Thus, more studies are needed to further elucidate this in the future.

## Data Availability

The raw data supporting the conclusions of this article will be made available by the authors, without undue reservation.

## References

[1] Alva R Mirza M Baiton A Lazuran L Samokysh L Bobinski A Oxygen toxicity: Cellular mechanisms in normobaric hyperoxia. *Cell Biol Toxicol.* (2023) 39:111–43. 10.1007/s10565-022-09773-7 36112262 PMC9483325

[2] Rao T Zhou Y Chen C Chen J Zhang J Lin W Recent progress in neonatal hyperoxic lung injury. *Pediatr Pulmonol.* (2024) 59:2414–27. 10.1002/ppul.27062 38742254

[3] Chu D Kim L Young P Zamiri N Almenawer S Jaeschke R Mortality and morbidity in acutely ill adults treated with liberal versus conservative oxygen therapy (IOTA): A systematic review and meta-analysis. *Lancet.* (2018) 391:1693–705. 10.1016/S0140-6736(18)30479-3 29726345

[4] Wingelaar T van Ooij P van Hulst R. Oxygen toxicity and special operations forces diving: Hidden and dangerous. *Front Psychol.* (2017) 8:1263. 10.3389/fpsyg.2017.01263 28790955 PMC5524741

[5] Demchenko I Welty-Wolf K Allen B Piantadosi C. Similar but not the same: Normobaric and hyperbaric pulmonary oxygen toxicity, the role of nitric oxide. *Am J Physiol Lung Cell Mol Physiol.* (2007) 293:L229–38. 10.1152/ajplung.00450.2006 17416738

[6] Paul M Poyan Mehr A Kreutz R. Physiology of local renin-angiotensin systems. *Physiol Rev.* (2006) 86:747–803. 10.1152/physrev.00036.2005 16816138

[7] Gan P Liao W Linke K Mei D Wu X Wong W. Targeting the renin angiotensin system for respiratory diseases. *Adv Pharmacol.* (2023) 98:111–44. 10.1016/bs.apha.2023.02.002 37524485

[8] Han C Zhang P Liu Y Zheng J Liu K Wei D Changes in angiotensin II and angiotensin-converting enzyme of different tissues after prolonged hyperoxia exposure. *Undersea Hyperb Med.* (2017) 44:39–44. 10.22462/1.2.2017.7 28768084

[9] Zhang P Han C Zhou F Li L Zhang H Liu W. Renin-angiotensin system and its role in hyperoxic acute lung injury. *Undersea Hyperb Med.* (2016) 43:239–46. 27416692

[10] Pagano A Barazzone-Argiroffo C. Alveolar cell death in hyperoxia-induced lung injury. *Ann N Y Acad Sci.* (2003) 1010:405–16. 10.1196/annals.1299.074 15033761

[11] Han C Guan Z Zhang P Fang H Li L Zhang H Oxidative stress induced necroptosis activation is involved in the pathogenesis of hyperoxic acute lung injury. *Biochem Biophys Res Commun.* (2018) 495:2178–83. 10.1016/j.bbrc.2017.12.100 29269294

[12] Zhu Y Cui H Lv J Li G Li X Ye F Angiotensin II triggers RIPK3-MLKL-mediated necroptosis by activating the Fas/FasL signaling pathway in renal tubular cells. *PLoS One.* (2020) 15:e0228385. 10.1371/journal.pone.0228385 32134954 PMC7058379

[13] Dong X Fan J Lin D Wang X Kuang H Gong L Captopril alleviates epilepsy and cognitive impairment by attenuation of C3-mediated inflammation and synaptic phagocytosis. *J Neuroinflammation.* (2022) 19:226. 10.1186/s12974-022-02587-8 36104755 PMC9476304

[14] Ulutas Z Ermis N Ozhan O Parlakpinar H Vardi N Ates B The protective effects of compound 21 and valsartan in isoproterenol-induced myocardial injury in rats. *Cardiovasc Toxicol.* (2021) 21:17–28. 10.1007/s12012-020-09590-6 32648158

[15] Risberg J van Ooij P. Hyperoxic exposure monitoring in diving: A farewell to the UPTD. *Undersea Hyperb Med.* (2022) 49:395–413. 10.22462/07.08.2022.1 36446287

[16] Qin H Zhuang W Liu X Wu J Li S Wang Y Targeting CXCR1 alleviates hyperoxia-induced lung injury through promoting glutamine metabolism. *Cell Rep.* (2023) 42:112745. 10.1016/j.celrep.2023.112745 37405911

[17] Mantell L Lee P. Signal transduction pathways in hyperoxia-induced lung cell death. *Mol Genet Metab.* (2000) 71:359–70. 10.1006/mgme.2000.3046 11001828

[18] Wang M Zhang F Ning X Wu C Zhou Y Gou Z Regulating NLRP3 inflammasome-induced pyroptosis via Nrf2: TBHQ limits hyperoxia-induced lung injury in a mouse model of bronchopulmonary Dysplasia. *Inflammation.* (2023) 46:2386–401. 10.1007/s10753-023-01885-4 37556072 PMC10673969

[19] Ren Y Qin S Liu X Feng B Liu J Zhang J Hyperoxia can induce lung injury by upregulating AECII autophagy and apoptosis via the mTOR pathway. *Mol Biotechnol.* (2023) 66(11):3357–68. 10.1007/s12033-023-00945-2 37938537 PMC11549204

[20] Hurskainen M Mižíková I Cook D Andersson N Cyr-Depauw C Lesage F Single cell transcriptomic analysis of murine lung development on hyperoxia-induced damage. *Nat Commun.* (2021) 12:1565. 10.1038/s41467-021-21865-2 33692365 PMC7946947

[21] Bhandari V Choo-Wing R Lee C Zhu Z Nedrelow J Chupp G Hyperoxia causes angiopoietin 2-mediated acute lung injury and necrotic cell death. *Nat Med.* (2006) 12(11):1286–93. 10.1038/nm1494 17086189 PMC2768268

[22] Batra V Gopalakrishnan V McNeill J Hickie R. Angiotensin II elevates cytosolic free calcium in human lung adenocarcinoma cells via activation of AT1 receptors. *Cancer Lett.* (1994) 76:19–24. 10.1016/0304-3835(94)90129-5 8124662

[23] Griendling K Berk B Socorro L Tsuda T Delafontaine P Alexander R. Secondary signalling mechanisms in angiotensin II-stimulated vascular smooth muscle cells. *Clin Exp Pharmacol Physiol.* (1988) 15:105–12. 10.1111/j.1440-1681.1988.tb01051.x 3078271

[24] Sun W Wu X Gao H Yu J Zhao W Lu J Cytosolic calcium mediates RIP1/RIP3 complex-dependent necroptosis through JNK activation and mitochondrial ROS production in human colon cancer cells. *Free Radic Biol Med.* (2017) 108:433–44. 10.1016/j.freeradbiomed.2017.04.010 28414098

